# Antineoplastic effects of histone deacetylase inhibitors in neuroendocrine cancer cells are mediated through transcriptional regulation of Notch1 by activator protein 1

**DOI:** 10.1002/cam4.1151

**Published:** 2017-08-04

**Authors:** Samuel Jang, Haining Jin, Madhuchhanda Roy, Alice L. Ma, Shaoqin Gong, Renata Jaskula‐Sztul, Herbert Chen

**Affiliations:** ^1^ Howard Hughes Medical Institute Birmingham Alabama 35233; ^2^ Department of Surgery University of Alabama at Birmingham Birmingham Alabama 35233; ^3^ Department of Biomedical Engineering University of Wisconsin‐Madison Madison WI 53715

**Keywords:** Activator protein 1, anticancer drug, electrophoresis mobility shift analysis, histone deacetylase inhibitor, neuroendocrine cancer, Notch pathway, transcription promoter

## Abstract

Notch signaling is minimally active in neuroendocrine (NE) cancer cells. While histone deacetylase inhibitors (HDACi) suppress NE cancer growth by inducing Notch, the molecular mechanism underlying this interplay has not yet been defined. NE cancer cell lines BON, H727, and MZ‐CRC‐1 were treated with known HDACi Thailadepsin‐A (TDP‐A) and valproic acid (VPA), and Notch1 mRNA expression was measured with RT‐PCR. Truncated genomic fragments of the Notch1 promotor region fused with luciferase reporter were used to identify the potential transcription factor (TF) binding site. The key regulatory TF was identified with the electrophoretic mobility shift assay (EMSA). The effect of HDACi on Notch1 level was determined before and after silencing the TF. TDP‐A and VPA induced Notch1 mRNA in a dose‐dependent manner. A functional DNA motif at −80 to −52 from the Notch1 start codon responsible for the HDACi‐dependent Notch1 induction was identified. Mutation of this core sequence failed to induce luciferase activity despite HDACi treatment. EMSA showed the greatest gel shift with AP‐1 in nuclear extracts. Knockdown of AP‐1 significantly attenuated the effect of HDACi on Notch1 induction. Interestingly, AP‐1 transfection did not alter Notch1 level, suggesting that AP‐1 is necessary but insufficient for HDACi activation of Notch1. Therefore, AP‐1 is the TF that binds to a specific transcription‐binding site within the Notch1 promotor region to trigger Notch1 transcription. Elucidating the HDACi activation mechanism may lead to the development of novel therapeutic options against NE cancers and facilitate the identification of clinical responders and prevent adverse effects.

## Introduction

Neuroendocrine (NE) malignancies are a heterogeneous group of neoplasms that originate in various anatomical locations and include carcinoid, islet cell tumors, and medullary thyroid cancers 1, 2, 3. Other anatomically unusual locations have been described also presenting as second primary tumors 4. Patients with NE cancer often present with multiple liver metastasis 2, 5, 6, which portend a dismal survival rate 7, 8 and result in excessive hormone secretion that can cause debilitating symptoms such as uncontrollable diarrhea, flushing, skin rashes, and heart failure. Surgery is the only curative option for isolated tumors, but widespread metastasis or degree of hepatic involvement at presentation makes complete resections often impossible 9, 10. Other forms of therapy including chemoembolization, radioembolization, radiofrequency ablation, cryoablation, and chemotherapy show limited efficacy 11, 12, 13, 14, 15, 16, 17, 18, 19, 20, 21, 22, 23. Therefore, there is a critical need for new therapeutic approaches to advanced NE cancers.

In an effort to find new therapeutic approaches to alter the malignant NE phenotype by activating tumor suppressor genes, we have examined aspects of transcriptional regulation associated with NE neoplasia. We previously demonstrated that inhibition of histone deactylase activates Notch signaling and induces cell cycle arrest and apoptosis in NE cancers 24, 25, 26. Additionally, our results indicate exogenous Notch1 expression decreases NE tumor markers and suppress cancer cell growth 27, 28, 29. The promising therapeutic potential of HDAC inhibitors (HDACi) as anticancer agents recently led to the FDA approval of four HDACi to date (SAHA, FK‐228, PXD‐101, and LBH‐589) against T‐cell lymphoma and multiple myeloma 30. As epigenetic dysregulation seems to be an important driver of NE cancers, histone modifications are appealing as therapeutic targets 31. Indeed, FDA‐approved and pre‐clinically tested HDACi are effective against NE cancers, among other solid tumors, in in vitro and in vivo models 26, 32, 33, 34.

While Notch signaling is minimally active in NE cancer cells, and HDACi induce Notch1 to suppress NE cancer growth 24, 32, 35, the molecular mechanism underlying this interplay has not been determined. Here, we perform deletion and mutational analysis of the Notch1 gene promoter region to investigate its transcription regulation. Our results provide novel mechanistic information regarding HDACi‐induced Notch1 activation that may help clarify the role of Notch1 in the pathogenesis of NE cancers and possible new therapeutic targets.

## Methods

### Cell culture

BON cells, a human gastrointestinal carcinoid cell line, were obtained from Drs. B. Mark Evers and Courtney M. Townsend, Jr. (University of Texas Medical Branch, Galveston, TX) and were maintained in Dulbecco's modified Eagle medium‐nutrient mixture Ham's F‐12 K 1:1 (Invitrogen, Carlsbad, CA). H727 cells, a human bronchopulmonary carcinoid cell line, were purchased from American Type Culture Collection (Manassas, VA) and were maintained in RPMI1640 (Life Technologies, Grand Island, NY). MZ‐CRC‐1 cells, a human medullary thyroid cancer cell line, were provided by Dr. Barry Nelkin (Johns Hopkins, Baltimore, MD) and were maintained in DMEM/F‐12 medium (Life Technologies). All media were supplemented with 10% fetal bovine serum (Sigma‐Aldrich, St Louis, MO), 100 IU/mL penicillin (Invitrogen), and 100 *μ*g/mL streptomycin (Invitrogen). All cell lines were grown in a humidified atmosphere of 5% CO_2_ at 37°C.

### Plasmid construction and materials

Luciferase report plasmid pGL2‐Basic vector that contains 961 nucleotide of the Notch1 promoter region was a generous gift from Dr. Tohru Kiyono (National Cancer Center, Tokyo, Japan). Notch1 promoter DNA fragments were prepared from PCR and restriction sites XhoI and NcoI. Multiple cloning sites of the vector were used to clone Notch1 promoter fragments. Thailandepsin‐A (TDP‐A) was obtained from Dr. YiQiang Cheng's laboratory 36. VPA (2‐propylpentanoic acid) and dimethyl sulfoxide (DMSO) were purchased from Sigma‐Aldrich.

### Luciferase assay

TransIT‐2020 (Mirus, Madison, WI) was used to transfect the recombinant plasmids into BON cells. HDACi TDP‐A (2 nmol/L) and VPA (3 mmol/L) were added 24 h after transfection. The Cell Culture Lysis Reagent in the Luciferase Assay System (Promega; cat# E1501) was used to harvest cells 24 h after treatment, and the commercial kit was used to perform the luciferase assay. Luciferase activity was measured by Monolight 3010 luminometer (Analytical Luminescence Laboratory, San Diego, CA). Promoter activity of each construct is represented by relative light unit (RLU) normalized to DMSO control for each construct expressed in average ± standard error of the mean (SEM).

### Electrophoretic mobility shift assay

Biotin‐labeled probes were prepared with Pierce Biotin 3′ End DNA Labeling Kit (Cat# 89818) to label two complementary probes at 3′ end separately. The probes were annealed by boiling and cooling. For more information, please check the protocol from Pierce.

The nuclear extract was prepared by first culturing BON cells to 80% confluency. HDAC inhibitors TDP‐A (2 nmol/L) and VPA (3 mmol/L) were added for 24 h, collected, washed with 1x PBS, incubated with hypotonic buffer (10 mmol/L HEPES, 10 mmol/L KCl, 1.5 mmol/L MgCl_2_, pH 7.9) for 10 min and spun down at 16,000 g for 10 min. The pellet was incubated on ice for 30 min with extraction buffer (20 mmol/L HEPES, 0.42 mmol/L NaCL, 0.2 mmol/L EDTA, 1.5 mmol/L MgCL_2_, 25% Glycerol, pH 7.9) while vortexing every 10 min. After incubation, the sample was centrifuged for 30 min to collect the supernatant nuclear extract. The nuclear extract was incubated with the DNA probes for 20 min in room temperature according to the manufacture protocol from LightShift Chemiluminescent Electrophoretic mobility shift assay (EMSA) Kit (Cat# 20148). For the gel supershift assay, 1 *μ*L of AP1/c‐Jun antibody (Sigma‐Aldrich; cat# A5968) was added to the nucleoprotein, gently mixed, and incubated at room temperature for 20 min.

The samples were run at 100 V on 6% polyacrylamide gel in 0.5x TBE (445 mmol/L Tris, 445 mmol/L boric acid, 10 mmol/L EDTA, pH 8.0), after prerunning the gel in 0.5x TBE for 1 h, until the loading dye moving to the bottom of the gel. The gel was transferred to BrightStar‐Plus nylon membrane (Ambion, Foster City, California; cat# AM 10120). The transferred DNA was crossed linked to the membrane with UV light cross‐link wash, bound with streptavidin following the protocol by LightShift Chemiluminescent EMSA Kit, and read by the ChemiDoc XRS+ system (Bio‐Rad Laboratories, Hercules, CA).

### Quantitative real‐time PCR

AP1/c‐jun siRNA (150 nmol/L, SMARTpool: ON‐TARGETplus, Dharmacon, Lafayette, CO) was transfected with Lipofectamine RNAiMAX reagent to knock down the expression of the gene in neuroendocrine cancer cell lines. Nonspecific (NS) siRNA (Ambion; cat# AM4635) was used as control. The cell lines were treated TDP‐A (0–10 nmol/L) or VPA (0–5 mmol/L) for 24 h by itself and also after pretreatment with AP1/c‐jun siRNA or NS siRNA for 24 h. Additionally, the cell lines were transfected with 10 *μ*g AP1/c‐jun plasmid (TransOmic; Cat# TCH1203; sequence based on *c‐Jun* gene) using Lipofectamine 2000 reagent for 24 h.

Total RNA was isolated from cultured cells using RNeasy Plus Mini Kit (Qiagen, Valencia, VA). Total RNA concentration was determined by NanoDrop Lite spectrophotometer (ThermoScientific, Wilmington, DE). Complementary DNA was synthesized from 2 *μ*g of total RNA using iScript cDNA Synthesis Kit (Bio‐Rad Laboratories).

The mRNA expression levels of *AP1/c‐Jun* and *Notch1* were measured by quantitative real‐time PCR (qRT‐PCR). The sequences for the PCR primers for the genes of interest are listed in Table S1. The qRT‐PCR was performed in triplicate on CFX Connect Real‐Time PCR Detection System (Bio‐Rad Laboratories). The cycle numbers obtained at the log‐linear phase of the reactions for target genes were normalized to housekeeping gene *s27* from the same sample measured concurrently. Finally, the comparative cycle threshold (Δ*C*_t_) method was used to calculate relative expression levels of target genes and was presented as average ± standard error of the mean (SEM).

## Results

### HDAC inhibitors induce Notch1 mRNA in neuroendocrine cancer

**Figure 1:**
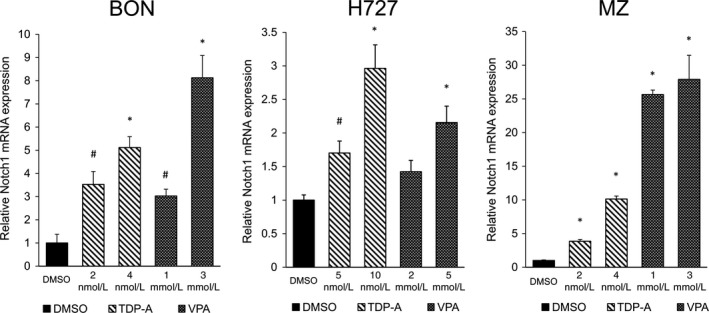
Notch1 mRNA expression after HDAC inhibitor treatment in neuroendocrine (NE) cell lines. Three NE cell lines BON, H727, and MZ‐CRC‐1 were treated with HDAC inhibitors TDP‐A and VPA in increasing concentrations close to their IC_50_. The data were plotted relative to the mRNA expression levels in cells treated with DMSO vehicle control. All values were presented as mean relative fold ± SEM (*P<0.01, ^#^ *P* < 0.05).

We first evaluated the efficacy of known HDACi Thailandepsin‐A (TDP‐A) and valproic acid (VPA) on their ability to induce Notch1 by the measurement of mRNA levels. TDP‐A is a recently reported HDACi produced by *Burkholderia thailandensis* isolated from the rice fields of Thailand with a promising anticancer efficacy at nanomolar concentrations 35, 36, 37. VPA is another well‐established HDACi approved by the FDA for the treatment of neuropsychiatric disorders. It exhibits potent anticancer activity, and it is currently under clinical trial for various cancers 38. As shown in Figure 1, TDP‐A and VPA significantly induced Notch1 mRNA expression in a dose‐dependent manner in BON, H727, and MZ cancer cell lines at concentrations close to their IC_50_. Highest induction levels were achieved in MZ cells where 4 nmol/L of TDP‐A and 3 mmol/L of VPA induced 10.1‐ and 27.9‐fold increases, respectively, in Notch1 gene expression compared to DMSO control. These data confirm the previous preclinical reports that HDACi induce the Notch1 pathway in NE cancers at the mRNA level.

### Identification of functional binding site in the Notch1 promoter

**Figure 2:**
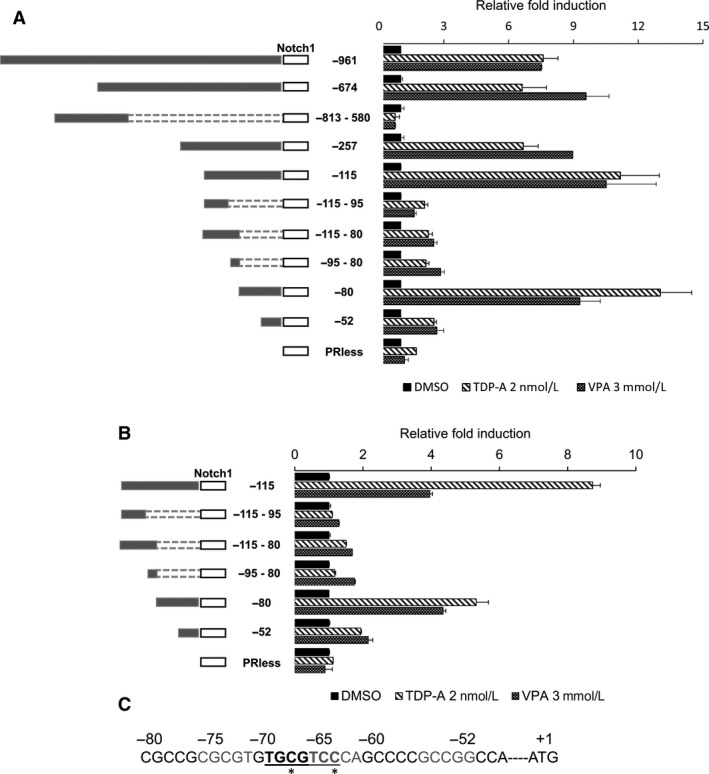
Deletion analysis of the human Notch1 promoter. BON (A) and H727 (B) cells were treated with TDP‐A and VPA after the cells were transfected with fragments of the Notch1 promoter region joined to a luciferase reporter. A schematic of the individual constructs is shown on the left. The promoter activities of the different deletion fragments were normalized to the relative light unit in cells treated with DMSO vehicle control for each individual constructs. All values were presented as mean relative fold ± SEM. (C) The active DNA sequence of the Notch1 promoter fragment (−80/−1) with the potential transcription factor binding site. The proposed noncanonical AP‐1 binding site is bolded and underlined. Sequences that are different from the canonical AP‐1 binding site are denoted with *.

The human Notch1 gene is located at chromosome 9q34.3. The region of the Notch1 promoter that is 5′ upstream from ‐961 to ‐1 position was cloned in pGL2‐basic plasmid vector upstream of the luciferase reporter gene. Luciferase reporter constructs harboring various 5′ and 3′ deletion fragments of the promoter in the same orientation were designated as −961/−1, −647/−1, −813/−580, −257/−1, −115/−1, −115/−95, −115/−80, −95/−80, −80/−1, and −52/−1. The promoterless‐pGL2 construct was used as control. The assigned numbers corresponded to the position with respect to the transcription start site as +1. A schematic diagram of the constructs is shown in Figure 2. The promoter activity of these constructs was measured in NE cancer cells BON (Fig. 2A) and H727 (Fig. 2B). The different promoter constructs were transiently cotransfected with GFP (internal control) into BON and H727 cells to verify no differences in transfection efficiency. After 24 h of incubation, these cells were treated with DMSO, 2 nmol/L TDP‐A, and VPA 3 mmol/L for 24 h. The promoter activities of these constructs are shown as fold changes of relative light unit (RLU) normalized to the DMSO control for each construct. As shown in Figure 2, there is no significant reduction in relative luciferase activity of constructs that extended to −1 from sites downstream of −52 with both TDP‐A and VPA treatments in BON and H727 cells. The robust luciferase activity with the −80/−1 promoter fragment and drastic reduction in promoter activity with the −52/−1 promoter fragment suggest that the potential transcription‐factor binding sites (TFBS) for HDACi‐induced Notch1 activation are in the −80/−52 promoter region. The DNA sequence of the potential TFBS is shown in Figure 2C. Additionally, the deletion constructs that do not include the potential TFBS (−813/−580, −115/−95, −115/−80, and −95/−80) showed a drastically reduced promoter activity.

**Figure 3:**
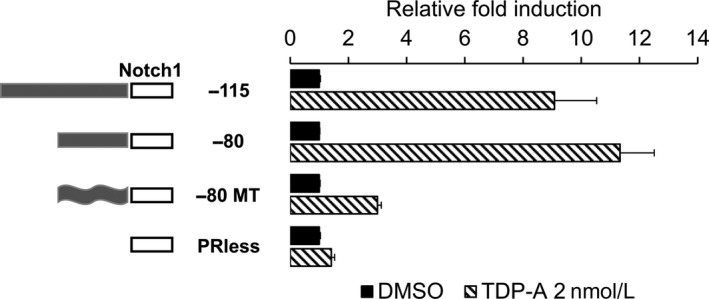
Mutational analysis of the potential transcription factor binding site (TFBS) on the Notch1 promoter. Sequences of the potential TFBS were mutated using site directed mutagenesis. The luciferase reporter plasmid containing the −115/−1, −80/−1, mutant (MT) −80/−1 sequences and promoterless fragment were transiently transected in BON cells and then treated with TDP‐A 2 nmol/L. A schematic of the individual constructs is shown on the left. The promoter activities of the different fragments were normalized to the relative light unit in cells treated with DMSO vehicle control for each individual constructs. All values were presented as mean relative fold ± SEM.

Once the potential TFBS were identified in the −80/−52 Notch1 promoter region, we used mutational analysis of the promoter coupled with the luciferase reporter assay to confirm and more accurately determine more accurately the active promoter region. In silico examination of the nucleotide sequence between residues −80 and −52 with the online bioinformatics tools TRANSFAC and MEME genomics suggested a potential noncanonical activating protein‐1 (AP‐1) binding site. Mutating the nucleotides in the potential AP‐1 binding site (−80‐CGCCGCGCGTGTGCGTCC‐63 to CGTTAATAGTAATTCC) markedly reduced the promoter activity of the −80/−1 fragment (Fig. 3). Together, these results suggest that the −80/−52 sequence of the Notch1 promoter is responsible for Notch1 induction by HDACi and that AP‐1 is the potential transcription factor that binds this region to activate it.

### Confirmation of transcription‐binding factor by EMSA

**Figure 4:**
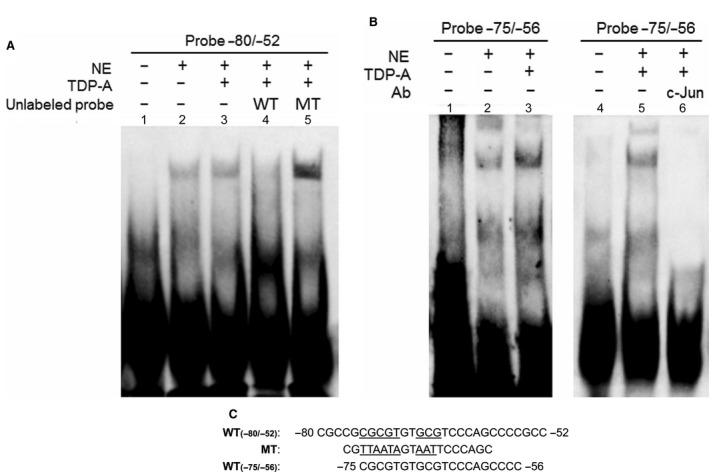
Binding of BON nuclear proteins to the active Notch1 promoter region by EMSA. Biotinylated double‐stranded oligonucleotides covering the promoter region −80/−52 (A) and −75/−56 (B) were incubated with BON nuclear extracts (lane A2–5; lane B2, 3, 5, 6). Biotinylated oligonucleotide without nuclear extracts was included for control (lanes A1, B1, and B4). (C) DNA sequences of −80/−52 wild type, −80/−52 mutant, and −75/−56 oligonucleotides are shown. Competing unlabeled oligonucleotide was used in lane A4 (−80/−52 wild type) and lane A5 (−80/−52 mutant). Lane B6 included 1 *μ*L of mouse polyclonal anti‐c‐Jun antibody. The biotinylated oligonucleotide was detected using chemiluminescent EMSA kit. NE, nuclear extract, WT, wild type, MT, mutant.

To verify whether the region of the Notch1 promoter between nucleotides −80 and −52 is a site for binding transcription factors, the DNA‐protein binding EMSA was performed in BON cells with a biotinylated 29‐bp oligonucleotide containing the sequence of the −80/−52 region of the Notch1 promoter. A competitive‐binding EMSA was performed to demonstrate whether unlabeled oligonucleotides with the wild‐type sequence and the mutated sequence used in the luciferase assay could compete with transcription factor binding for the potential TFBS. The results indicated that this region binds nuclear proteins isolated from both DMSO control and TDP‐A treated NE cancer cells (Fig. 4A, lanes 2 and 3). Additionally, the specific binding activity of nuclear extracts could be competed at 100‐fold excess with the unlabeled wild type −80/−52 probe (Fig. 4A, lane 4), but not with the mutant probe (Fig. 4A, lane 5). These results agree with those derived from the luciferase assays and confirm the identification of a Notch1 transcription regulatory element.

We further investigated the specificity of the proteins binding to this region by performing EMSA with a shorter 20‐bp oligonucleotide containing the sequence of the −75/−56 region that more closely resembled the potential AP‐1 binding site. The shorter probe also bound to the nuclear proteins from NE cancer cells with and without TDP‐A treatment (Fig. 4B, lane 2 and 3). It should be noted that AP‐1 is a complex of gene products from the Jun and the Fos subfamilies, and the role of its components in cancer biology has been extensively studied (see Discussion). The increasing literature on the tumor suppressive role of c‐Jun 39, its association with HDAC 40, and the higher nuclear expression pattern of c‐Jun compared to other AP‐1 members in human NE tumors^41^ prompted us to select c‐Jun for further binding studies. To investigate whether AP‐1 is the transcription factor that binds to the target site, nuclear extracts from NE cancer cells treated with TDP‐A were incubated with anti‐c‐Jun antibody prior to detection of DNA‐protein by EMSA with the radiolabeled −75/−56 oligonucleotide. Antibody against c‐Jun induced a super shift of the DNA probe (Fig. 4B, lane 6), supporting that AP‐1, with c‐Jun as a component, is the transcription factor that binds to the Notch1 promoter after HDACi treatment.

### Knockdown of AP‐1 reduced Notch1 mRNA expression

**Figure 5:**
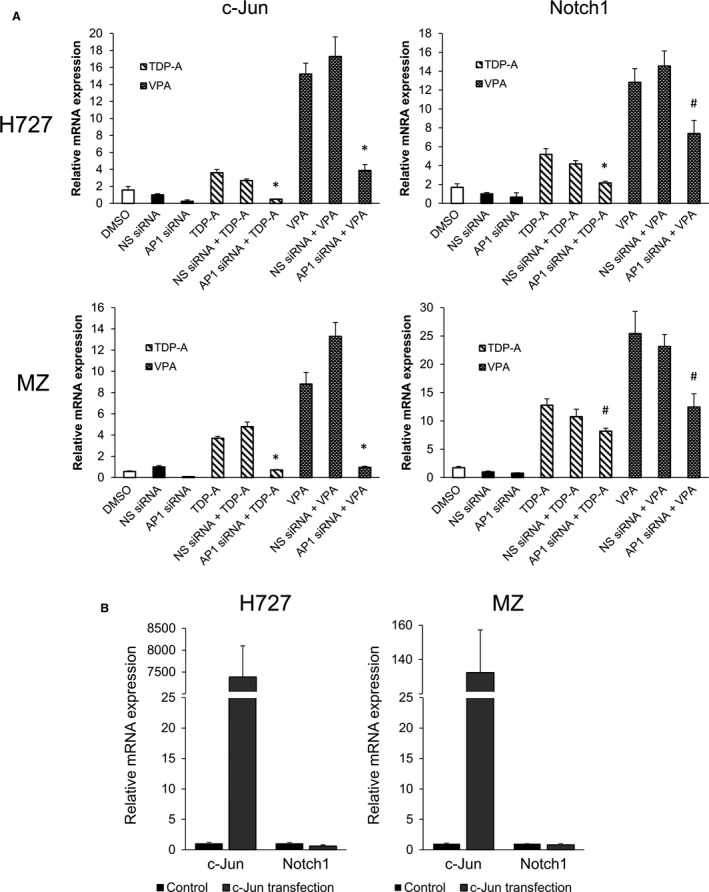
Effect of AP‐1/c‐Jun knockdown and overexpression on c‐Jun and Notch1 mRNA transcription. (A) H727 and MZ cells were transiently transfected with either c‐Jun or nonspecific (NS) control siRNA. Twenty‐four hours later, the cells were then treated with TDP‐A (10 nmol/L in H727, 5 nmol/L in MZ) and VPA (5 mmol/L in H727, 3 mmol/L in MZ) close to their IC_50_ for 24 h. The data were plotted relative to the mRNA expression levels measured by RT‐PCR in cells treated with NS control siRNA. The mRNA expression of cells treated with DMSO only is also shown. Statistical significance was noted with (*P < 0.01) and (^#^ *P* < 0.05) when compared against cells transfected with NS siRNA and subsequently treated with the HDAC inhibitors. (B) H727 and MZ cells were transiently transfected with plasmids containing the gene sequence of c‐Jun (DNA concentration: 10 *μ*g). The data were plotted relative to the mRNA expression levels in cells treated with DMSO vehicle control. All values were presented as mean relative fold ± SEM.

Since our results showed that AP‐1 associates with the active site of the Notch1 promoter and that c‐Jun is a component of the AP‐1 complex, we examined the effect of knocking down c‐Jun gene on Notch1 expression using RT‐PCR. H727 and MZ cells were transfected with AP‐1/c‐Jun siRNA or nonspecific (NS) siRNA for 24 h. AP‐1/c‐Jun siRNA transfection achieved greater than fourfold decrease in its mRNA expression in MZ cells and greater than 10‐fold decrease in H727 cells compared to NS siRNA control. The AP‐1/c‐Jun and NS siRNA transfected cells were treated with TDP‐A or VPA with doses close to their IC_50_. While the HDACi treatments induced c‐Jun mRNA expression in the control groups (no transfection and NS siRNA transfection), knockdown of AP‐1/c‐Jun significantly attenuated the increase in expression by HDACi (Fig. 5A). Similarly and more importantly, knockdown of AP‐1/c‐Jun resulted in significant suppression of Notch1 mRNA induction by both TDP‐A (*P* = 0.008 in H727, *P* = 0.022 in MZ) and VPA (*P* = 0.028 in H727, *P* = 0.028 in MZ).

To further investigate the effect of c‐Jun overexpression on Notch1, H727 and MZ cells were transfected with a plasmid containing the gene sequence of *c‐Jun*. The transfection, somewhat remarkably, resulted in over 7000‐fold increase in relative c‐Jun mRNA level in H727 cells and over 130‐fold increase in MZ cells, where the difference may be attributed to transfection efficiencies of the specific cell lines (Fig. 5B). However, the massive induction of c‐Jun resulted in no significant change in Notch1 mRNA expression. Together these results reveal that AP‐1 modulates Notch1 expression in response to the disruption of histone deacetylation via an upstream mRNA transcription regulatory element, and that c‐Jun plays an obligatory role in this antineoplasia mechanism.

## Discussion

There is an urgency to find new therapeutic options for NE cancers given the current lack of effective treatments. Octreotide has been effective for treating low‐grade and functioning NE tumors; however, the patients develop resistance over time. Alternatively, *β*‐emitting radionuclide conjugated octreotide have not shown consistent clinical benefit 42. IFN‐*α* is approved by the European Medicines Agency for treating high‐grade NE tumors, but it has severe side‐effects 43. Tyrosine kinase inhibitors are under clinical evaluation^44^ and several small molecules that target IGF1R or VEGFR/PDGFR are also being evaluated 43. Other targeted therapies have also been studied, but the efficacies were limited 45, 46.

We have previously demonstrated that Notch1 acts as a tumor suppressor in NE cancers, as the overexpression of Notch1 in carcinoid and medullary thyroid cancer cell lines at the transcriptional level resulted in inhibition of NE cancer cell growth and suppression of NE tumor markers and hormones 28, 29, 47. We identified that drugs that inhibit histone deactylase activity induce Notch1 in NE cancer cells and show promising anticancer activities. In the studies that examine genetic and pharmacologic induction of Notch1, there was a close correlation between Notch1 mRNA and protein expression 25, 28, 32, 48, 49. Our group conducted a pilot phase II clinical trial where we administered VPA 500 mg daily to eight patients with low‐grade pancreatic NE cancer and midgut carcinoids 45. Not only did the VPA treatment lead to partial response and disease stabilization, but also the measurement of Notch1 mRNA level in pre and posttreatment biopsy samples showed greater than 10‐fold induction. Importantly, Notch1 upregulation was associated with improved outcomes. To further elucidate the mechanism of this therapy, in this study, we identified the functional region of the Notch1 promoter in the context of HDACi treatment of NE cancers. The results revealed that the transcription factor AP‐1 associates with the binding site −75‐CGCGTGTGCGTCCCAGCCCC‐56 and activates Notch1 transcription upon HDACi treatment. Additionally, depletion of AP‐1 using siRNA resulted in the suppressed ability of HDACi to induce Notch1 transcription.

Since its discovery in 1987 50, the transcription factor AP‐1 has been extensively studied in the context of cell proliferation, apoptosis, oncogenesis, and metastasis. As previously mentioned, it is a dimeric protein complex whose major components are the Jun (c‐Jun, JunB, and JunD) and the Fos (c‐Fos, FosB, Fra1, and Fra2) subfamilies. Not only are its members expressed in diverse cell types and tissues, but AP‐1 binding sites are also ubiquitously expressed in a wide range of promoter and enhancer regions 51. AP‐1 has basal gene expressing activity, and it is inducible by multiple stimuli including growth factors, proinflammatory cytokines, and UV radiation 52. Initial in vitro studies demonstrated that AP‐1 has the highest affinity for a heptamer consensus sequence 5′‐TGA(C/G)TCA‐3′ known as 12‐O‐tetradecanoylphobol‐13‐acetate responsive element (TRE) 53. However, numerous other noncanonical but functional AP‐1 binding sites with similar sequences have been identified 54, 55. The proposed AP‐1 binding site on the Notch1 promoter (bolded and underlined in Fig. 2C) differs in two motifs from that of TRE (5′‐TGCGTCC‐3′). The sequences to which AP‐1 binds may be influenced by the constituent isoforms of the dimeric protein and their interactions with other regulatory proteins.

The complexity of AP‐1 regulation led to the conclusion that the functions of AP‐1 and its component proteins heavily depend on the specific cell type and the context in which they are expressed 39. While initial observations suggested that c‐Jun acts mainly as an oncogene and JunB as a tumor suppressor, this model is being challenged in light of new findings. For example, c‐Jun potently induces apoptosis in UV‐irradiated cells, it is involved in DNA repair, and it upregulates expression of tumor suppressor p14/p19 39. AP‐1 has been reported to act as a tumor promoter or a tumor suppressor, depending on the cellular context 56. Here, we report that AP‐1 acts as a tumor suppressor by inducing Notch1 in NE cancer cells that are treated with HDACi.

The role of AP‐1 in NE cancers has been examined previously by immunohistochemistry staining a cohort of human colorectal NE tumors 41. Scoring of the staining intensity of AP‐1 family proteins revealed that c‐Jun, JunB, Fra‐1, and Fra‐2 were elevated in NE cancers. While c‐Jun exhibited a stronger nuclear straining, JunB was more prevalently localized in the cytoplasm, suggesting that c‐Jun may have a more important role in regulating transcriptional activities in NE cancers. Interestingly, c‐Jun overexpression occurred more frequently in low‐grade NE tumors compared to those that were high grade. We previously showed that elevations in Notch isoforms portend a less aggressive tumor phenotype and favorable clinical outcomes in cancer patients where Notch induction is therapeutic 27, 57, 58. Similarly, elevations of AP‐1 and c‐Jun may be protective mechanisms upstream of Notch, which can also determine NE cancer aggressiveness.

The observation that the AP1 family protein c‐Jun activates Notch1 transcription has recently been made in triple‐negative breast cancer cell lines 59, therefore, consistent with our data. In similar experiments as ours, Xie et al. showed that silencing c‐Jun with siRNA led to the decreased Notch1 mRNA expression and reduced luciferase activity of the Notch1 promoter. The authors also showed that c‐Jun N‐terminal kinase (JNK) phosphorylates c‐Jun to activate it, which induced Notch1 expression. They proposed that the activated c‐Jun binds to the Notch1 promoter, but did not provide any data to support the hypothesis. Although Notch signaling is involved in driving tumorigenesis in breast cancers among other cancers, it is well known that Notch signaling exerts both oncogenic and tumor suppressive functions depending on the cellular context 60. The tumor suppressive role of the Notch pathway has been observed in NE 27, 28, thyroid 57, 58, brain 61, hepatocellular, head and neck, skin, and various blood cancers 62. The finding that c‐Jun plays a role in the transcription of Notch1 in both types of cancers where high Notch1 expression is either oncogenic or tumor suppressive suggests that the Notch1 induction pathway may be conserved. Identification of regulators of AP‐1/c‐Jun expression or specific downstream effects of Notch1 induction in individual cellular contexts may discern its oncogenic or tumor antiproliferative effects.

It will be beneficial to further investigate Notch1 upstream regulators that contribute to specific HDAC‐dependent control signaling. A direct clinical application of the discovery of the AP‐1 binding site on the Notch1 promoter in the context of HDACi treatment is the identification of clinical nonresponders before the initiation of HDACi therapy. In the era of personalized medicine, next‐generation sequencing of tumor biopsy samples can be used to verify that the Notch1 promoter region does not contain mutations that can hinder the action of HDACi. The question remains, however, how is AP‐1 regulated in NE cancer cells and how does HDACi activate AP‐1 or its family member proteins to induce Notch1 expression? Our data show that c‐Jun is necessary, but simply the endogenous presence or the overexpression of c‐Jun is insufficient to drive Notch1 activity (Fig. 5B); it needs the activation provided by HDACi. Additionally, given the redundancy of the proteins that make up AP‐1, it is possible that other AP‐1 family member proteins can activate Notch1, with or without interactions with other proteins. It is commonly known that phosphorylation of c‐Jun at different sites by numerous kinases can activate its transcription 39, 40, 63, and perhaps HDACi play a role in the signaling cascades. Interestingly, it has been shown in transfected human embryonic kidney cells that HDAC3 inhibits the transcriptional activity of c‐Jun by direct binding and that phosphorylation of c‐Jun by JNK relieves the inhibition 40. Both TDP‐A and VPA are class I HDACi which can bind and potentially deactivate HDAC3. Another possible mechanism is an epigenetic model where HDACi could induce the acetylation of histone in the region of the Notch1 promoter by altering the chromatin structure and make the DNA fragment accessible to AP‐1.

An understanding of which regulatory elements are involved in the HDACi activation of AP‐1/c‐Jun and Notch1 is necessary to further understand the biology of NE cancer and the mechanism of HDACi therapy, to identify clinical responders, and to elucidate potential candidates of drug targeting and combination therapies.

## Conflict of Interest

There are no conflict of interest disclosures from any authors.

## Supporting information


**Table S1.** Primer sequences for qRT‐PCR.
