# Supplementary material for: Antineoplastic effects of histone deacetylase inhibitors in neuroendocrine cancer cells are mediated through transcriptional regulation of Notch1 by activator protein 1
Source: Cancer Med. 2017 Aug 4;6(9):2142–52. doi: 10.1002/cam4.1151 (PMC5603840; doi:10.1002/cam4.1151)
Supplement: Supplementary file 1 — Table S1. Primer sequences for qRT‐PCR. [file CAM4-6-2142-s001.docx]

**Antineoplastic Effects of Histone Deacetylase Inhibitors in Neuroendocrine Cancer Cells are Mediated through Transcriptional Regulation of Notch1 by Activator protein 1 (AP-1)**

Samuel Jang, Haining Jin, Madhuchhanda Roy, Alice L Ma, Renata Jaskula-Sztul, Herbert Chen

Table S1. Primer sequences for qRT-PCR

| AP1/c-Jun | Forward primer: 5′-CCT TGA AAG CTC AGA ACT CGG AG-3′ |
| --- | --- |
|  | Reverse primer: 5′-TGC TGC GTT AGC ATG AGT TGG C-3′ |
| Notch1 | Forward primer: 5′-CAT GCT GGA GGA CCT CAT CA-3′ |
|  | Reverse primer: 5′-GGC TCC GTT CTT CAG GAG CA-3′ |
| S27 | Forward primer: 5′-TCT TTA GCC ATG CAC AAA CG-3′ |
|  | Reverse primer: 5′-TTT CAG TGC TGC TTC CTC CT-3′ |
